# 

**Published:** 1892-11

**Authors:** 


### CONTENTS:

ORIGINAL ARTICLES: PAGE

The Use of Mallein for the Diagnosis of Glanders in Horses and Experiments with an Albumose Extracted from Cultures of the Bacillus Malleus. By E. A. de Schweinitz, Ph.D., and F. L. Kilborne, B.V.S.... .......... 643
Food Inspection. By Olaf Schwarzkopf....... ......... .................... 658
Processes of Refrigeration Used for Dressed Beef in Transportation, etc. By Williamson Bryden, V.S.................................................. 666
Abortion. By R. G. Webster, V.M.D.......................................... .. 670
Local Tuberculous Abscess in a Bull. By Wyatt Johnston, M.D............ 673
The Etiology of Southern Cattle Plague—Texas Fever. By F. S. Billings.... 676
The Scientific Investigations of the Bureau of Animal Industry. By Dr. D. E. Salmon............................................ ..................... 692

EDITORIAL:

Veterinary Colleges—They Come...................... ...... ........... .... 719
New York State and Tuberculosis in Cattle of the United States............... 721

SOCIETY PROCEEDINGS:

Keystone Veterinary Medical Association.............................. .. .... 723
Keystone Veterinary Medical Association.... ........... ................. 723

SOCIETY MEETINGS:

Iowa State Veterinary Medical Association.................................. 724
Illinois State Veterinary Medical Association................................ 724

